# Constructing and applying a tiered family engagement strategy for managing neurogenic bowel dysfunction in TSCI patients

**DOI:** 10.3389/fneur.2025.1658921

**Published:** 2026-01-14

**Authors:** Hongyan Li, Zhaoxuan Wang, Jia Ding, Qian Wang, Ruiling Wang

**Affiliations:** Third Hospital of Hebei Medical University, Shijiazhuang, China

**Keywords:** caregiver burden, family-centered rehabilitation, health education, neurogenic bowel dysfunction, personalized care, traumatic spinal cord injury

## Abstract

**Background:**

Neurogenic bowel dysfunction (NBD) severely impairs life quality after thoracic spinal cord injury (TSCI). In this 133-patient trial, we evaluated whether a literacy-adaptive, family-centred programme improves outcomes beyond guideline care.

**Methods:**

In this single-centre, prospective, stratified, randomised controlled trial, 137 T6–T12 TSCI patients and their primary caregivers were assigned to routine follow-up (*n* = 69) or a tiered family-engagement intervention matched to caregiver educational level (*n* = 68). Primary endpoint was change in NBD score; secondary endpoints were caregiver execution score, Zarit Burden Interview (ZBI) and SF-36. Assessments occurred at discharge, 6 and 12 months (intention-to-treat).

**Results:**

At 12 months, the intervention group showed a larger mean NBD reduction than controls (3.5 ± 4.8 vs. 2.6 ± 5.2) and greater improvements in execution (*d* = 1.55), ZBI (*d* = −1.33) and SF-36 (*d* = 0.88). Benefits increased step-wise from tertiary- to primary-education strata, with no significant effect among highly educated caregivers. No programme-related adverse events occurred.

**Conclusion:**

Tailoring NBD management to caregiver literacy yields clinically meaningful, durable gains in bowel function, caregiving competence, burden and patient quality of life, especially when baseline self-efficacy is low. Literacy-stratified discharge pathways should be integrated into rehabilitation practice.

## 1 Introduction

**Figure 1:**
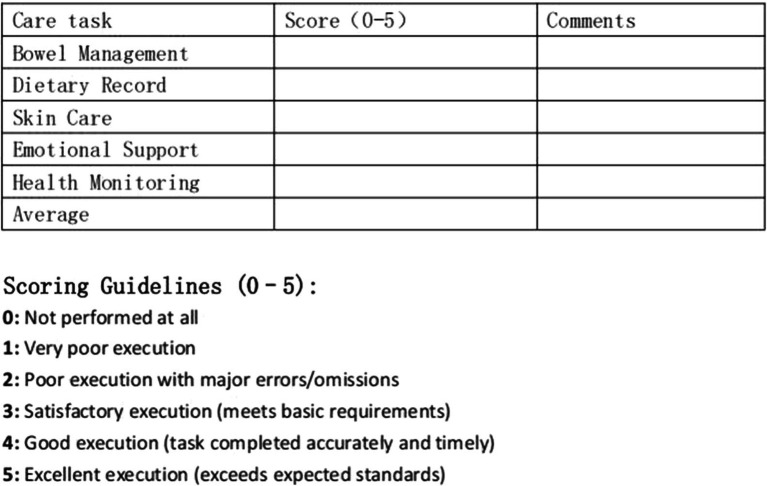
Caregiver Execution Status Assessment Tool. The table evaluates five core care domains (Bowel Management, Dietary Record, Skin Care, Emotional Support, and Health Monitoring) using a 6-point scale (0-5), where 0 indicates task not performed, 1 indicates very poor execution, 2 indicates poor execution with major errors/omissions, 3 indicates satisfactory execution meeting basic requirements, 4 indicates good execution with accurate and timely completion, and 5 indicates excellent execution exceeding expected standards. An average score across all domains provides an overall assessment of caregiver competency.

**Figure 2:**
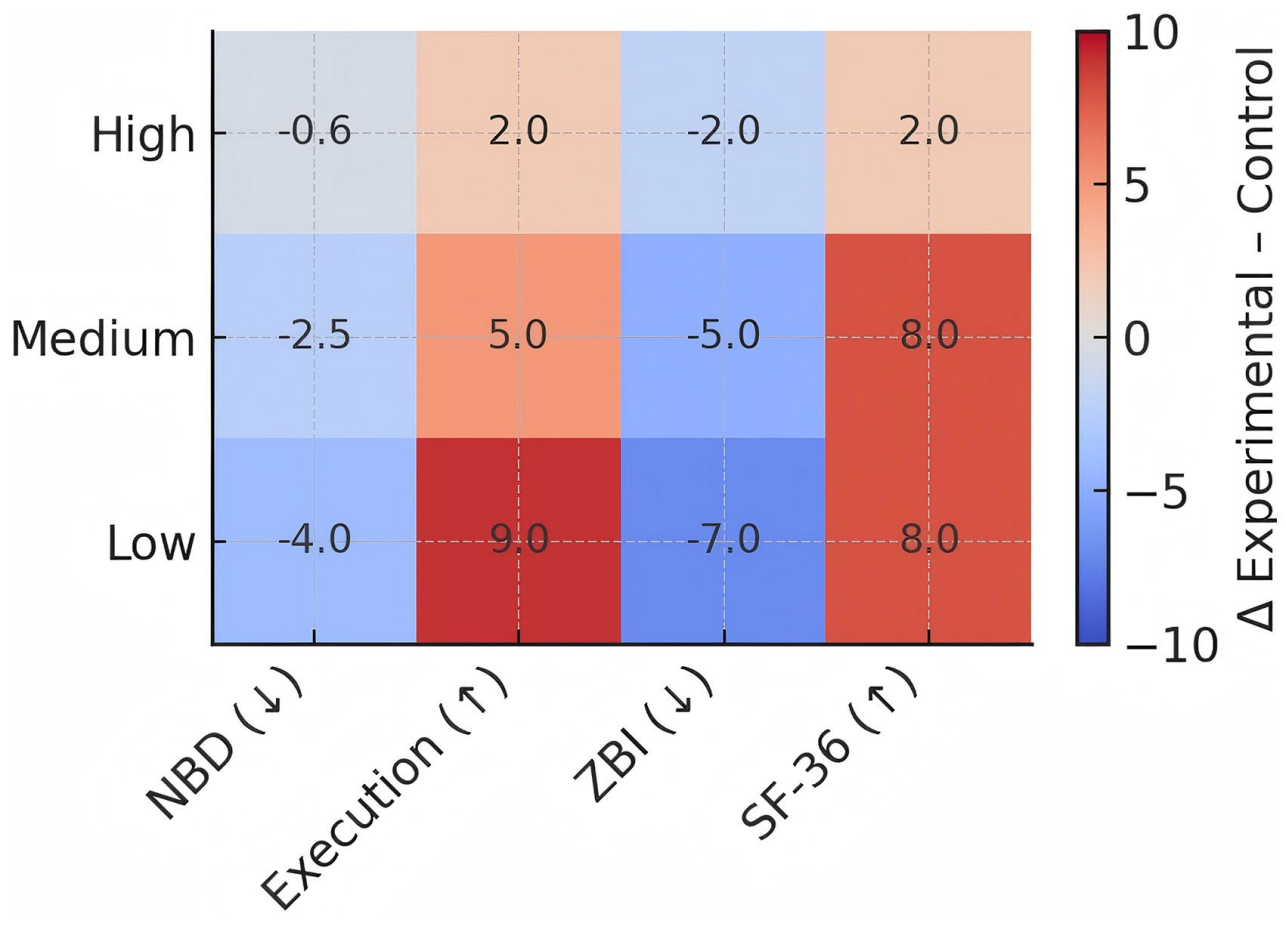
Heatmap comparing changes between experimental and control groups across four outcome measures (NBD score, Execution status, ZBI score, and SF-36) stratified by caregiver education level (High, Medium, Low). Color gradient ranges from blue (-10) representing negative changes to red (+10) representing positive changes, with white (0) indicating no change. Values represent the magnitude of difference between groups at 12-month follow-up.

**Figure 3:**
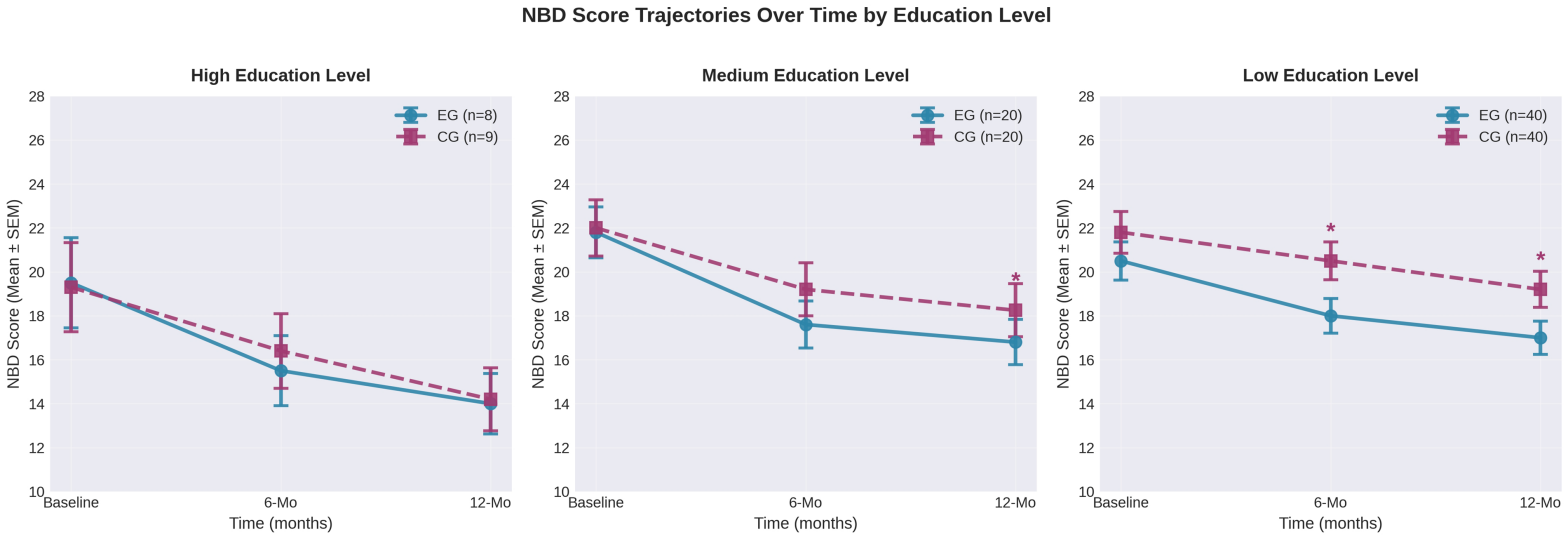
NBD score trajectories over time stratified by caregiver education level. The figure displays three panels showing changes in NBD scores from baseline through 6 and 12 months for experimental group (EG, solid blue line) and control group (CG, dashed red line) across high, medium, and low education levels. Error bars represent standard error of the mean. Asterisks denote statistical significance (*p < 0.05) between groups at each timepoint.

**Figure 4:**
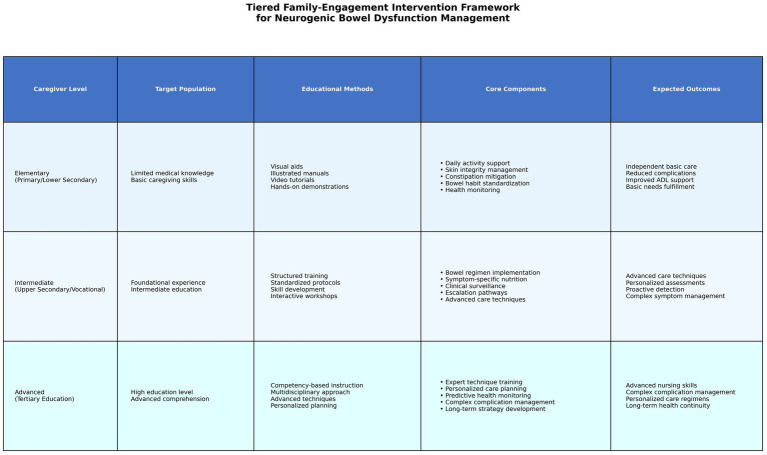
Tiered family-engagement intervention framework for neurogenic bowel dysfunction management. The framework delineates three caregiver competency levels (Elementary, Intermediate, and Advanced) with corresponding target populations, educational methods, core intervention components, and expected outcomes. Elementary level targets caregivers with primary/lower secondary education using visual aids and hands-on demonstrations; Intermediate level targets those with upper secondary/vocational education using structured training and interactive workshops; Advanced level targets those with tertiary education using expert technique training and personalized care planning.

**Figure 5:**
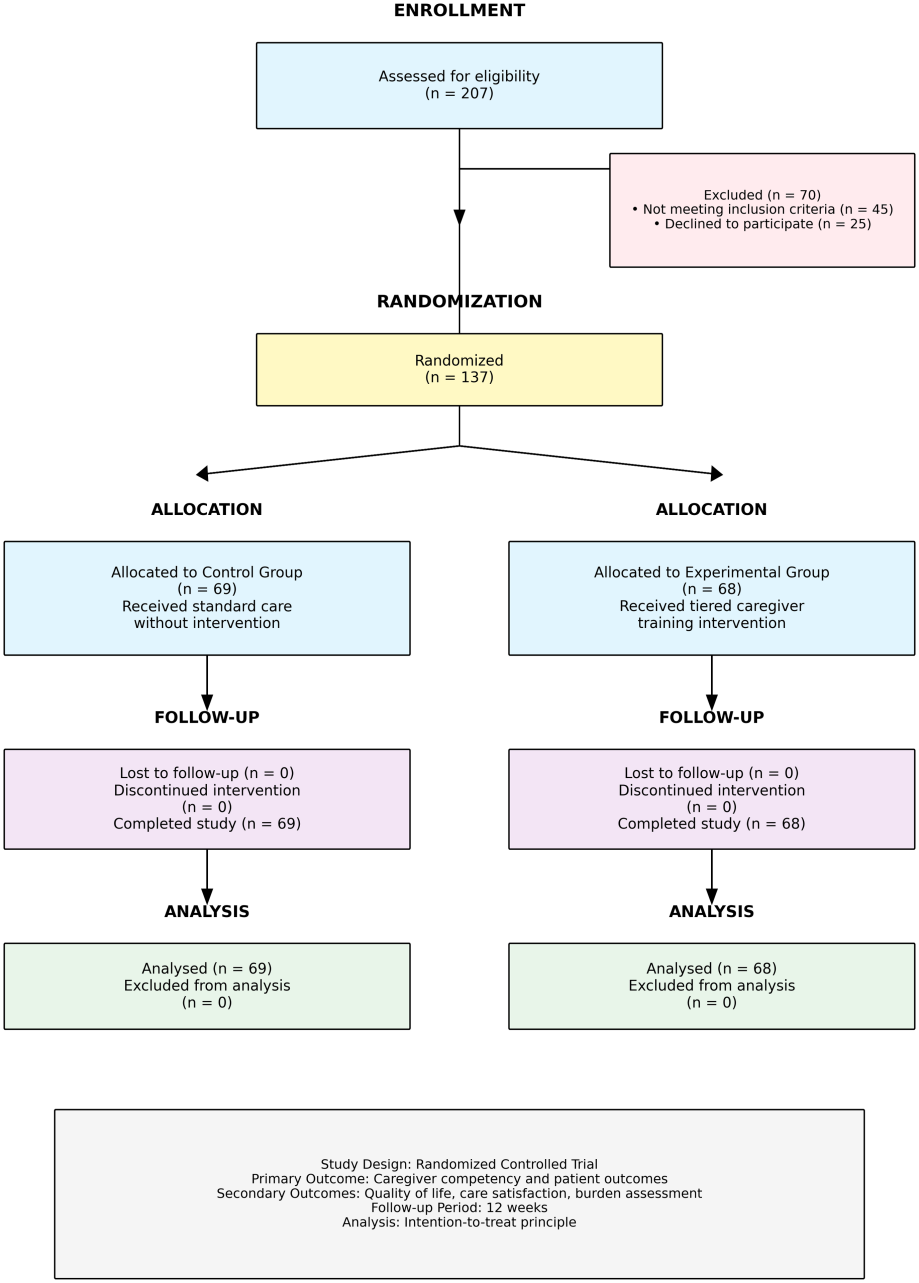
CONSORT flow diagram depicting participant progression through the randomized controlled trial. From 207 patients assessed for eligibility, 70 were excluded (45 not meeting inclusion criteria, 25 declined to participate), resulting in 137 randomized participants. Allocation assigned 69 to control group (standard care) and 68 to experimental group (tiered caregiver training intervention). Zero participants were lost to follow-up or discontinued intervention in either group. Final analysis included all randomized participants (69 control, 68 experimental) using intention-to-treat approach. Primary outcome: caregiver competency and patient outcomes; Secondary outcomes: quality of life, care satisfaction, burden assessment. Follow-up period: 12 weeks.

Traumatic spinal cord injury (TSCI) has increasingly become a critical global health priority in recent decades (1, 2). Epidemiological surveillance reveals a progressive rise in TSCI incidence worldwide (3, 4), with China demonstrating a national prevalence of approximately 0.06%, primarily attributable to falls from heights and traffic accidents (5). Anatomically, thoracic spinal cord injuries constitute 35–45% of all spinal cord injuries, of which 50–60% involve the lower thoracic segments (T7–T12) (6, 7). A particularly prevalent complication, neurogenic bowel dysfunction (NBD), affects over 80% of TSCI patients, clinically manifesting as constipation, abdominal distension, prolonged defecation duration, and impaired defecatory reflexes. These gastrointestinal impairments profoundly disrupt patients’ daily functioning, diminish quality of life metrics, and create substantial barriers to societal reintegration, collectively imposing significant socioeconomic burdens on affected families, healthcare infrastructures, and broader communities (8, 9) (see Figures 1–5).

Current clinical research predominantly emphasizes acute-phase gastrointestinal symptom management, despite evidence showing Chinese thoracic TSCI patients experience extended hospitalization periods (mean 32.4 ± 37.7 days) (10). Post-discharge rehabilitation typically transitions to family-mediated care, yet critical gaps persist: most caregivers lack standardized training in medical knowledge and procedural competencies. This deficiency becomes particularly evident when managing complex neurogenic bowel pathologies, where inadequate informational resources and improper technique implementation frequently converge, ultimately compromising care efficacy (11).

Despite the high prevalence of NBD and its impact on TSCI patient outcomes, three critical gaps persist in current management approaches. First, existing caregiver education programs deliver uniform content regardless of health literacy levels (12, 13), ignoring evidence that educational attainment fundamentally shapes information processing and skill acquisition (14, 15). Second, no published studies have systematically stratified post-discharge interventions by caregiver competency, missing opportunities to optimize resource allocation. Third, whether tailoring intervention complexity to caregiver literacy translates into measurable improvements in patient bowel function, caregiver burden, and quality of life remains unknown.

We hypothesized that a literacy-adaptive, tiered family-engagement intervention would produce superior outcomes compared to guideline-based care alone, with benefit magnitude inversely proportional to caregiver educational attainment. This prospective, stratified randomized controlled trial aimed to: (1) compare the effectiveness of tiered versus standard NBD management across three caregiver education strata; (2) quantify intervention effects on patient bowel function (NBD score), caregiver execution competency, caregiver burden (ZBI), and patient quality of life (SF-36); and (3) determine whether educational stratification identifies subgroups most likely to benefit from intensive family-centered interventions.”

## 2 Method

The study was approved by the Medical Ethics Committee of the Third Hospital of Hebei Medical University (Approval No. W2023-067-1). Written informed consent was obtained from all participants. Clinical Trial Registration: This study was not registered in a clinical trial database for the following reasons: (1) the intervention focuses on educational and behavioral caregiver training rather than experimental clinical treatment of patients; (2) primary outcomes measure caregiver performance, caregiver burden, and patient quality-of-life improvements resulting from enhanced home care rather than direct therapeutic effects; and (3) the study design meets exemption criteria for educational research established by international medical journal guidelines and ethics review standards. All regulatory ethical review processes were completed to ensure study transparency and compliance. We acknowledge that registration may enhance transparency and have discussed this limitation in the Limitations section. Between June 1, 2023, and April 30, 2024, 207 spinal cord injury (SCI) patients meeting the following criteria were enrolled from our institution: 1. Diagnosis confirmed by International Standards for Neurological Classification of Spinal Cord Injury (ISNCSCI) criteria, supported by clinical symptoms, medical history, physical signs, and radiological evidence. 2. Injury level T6-T12 with gastrointestinal dysfunction manifestations (e.g., abdominal pain, bloating). 3. Availability of a stable family caregiver (daily care provision ≥8 h). 4. Preserved cognitive capacity for verbal communication and willingness to participate. 5. ASIA grade II or III (patients in grades II or III share similar care plans, whereas those in grades I or IV are excluded). Patients were excluded for: 1. Non-SCI-related gastrointestinal dysfunction. 2. Prior gastrointestinal surgery. 3. Caregivers with physical/psychiatric comorbidities. 4. Surgical intervention for NBD during hospitalization. 5. Declined participation. After exclusions.137 participants were enrolled in the study. To ensure optimal baseline comparability between groups, participants were matched through stratified randomization based on age, sex, ASIA Impairment Scale (AIS) grade, caregiver education level, and admission NBD scores. Following this protocol, 69 patients were allocated to the control group and 68 to the experimental group. This study was conducted as a prospective controlled trial (Table 1).

**Table 1:** Basic information.

Characteristic categories	EG (*n* = 68)	CG (*n* = 69)
Sex (M/F)	35/33	35/34
Age (years)	40.1 ± 5.5	39.2 ± 4.3
Education level (low/med/high)	40/20/8	40/20/9
ASIA impairment scale		
B	20	19
C	48	50

EG, Experimental group; CG, Control group.

### 2.1 Intervention scheme

Following hospital admission, both groups received standardized interventions according to the 2022 gastrointestinal dysfunction management guidelines developed by the German Association of Scientific Medicine (DGWMI) (16). Diagnostic assessment: Comprehensive patient evaluation, including detailed anamnesis focusing on stool frequency, consistency, sensation of evacuation, diet, and medication use. Abdominal assessment, anorectal examination, and advanced diagnostic tools (e.g., ultrasound and colonoscopy) are employed for thorough evaluation. Bowel management: The goal is to achieve regular bowel movements and secondary continence. Specific measures include: Dietary management: ensuring a high-fiber diet and adequate fluid intake (1500-2000 mL/day), with a recommended fiber intake of 30 g/day (17, 18). Bowel techniques: physical methods such as abdominal massage, digital stimulation, and enemas, with bowel movements ideally scheduled post-meal to utilize the gastrocolic reflex. Pharmacological treatment: Use of oral and rectal laxatives (e.g., glycerin, bisacodyl) to regulate stool consistency. Physical therapy: implementation of pelvic floor exercises and biofeedback training, especially for patients with the ability to sense and control their pelvic floor muscles. Complication management: regular monitoring for complications such as autonomic dysreflexia, constipation, and flatulence, with adjustments made to the treatment plan to minimize health risks. The key elements of family education include: basic knowledge: educating family members about the basic concepts of NBD, its symptoms (e.g., constipation, fecal incontinence, bloating), and the goals of bowel management. Diet and medication management: teaching family members how to provide a high-fiber diet, ensure adequate fluid intake, and how to administer laxatives and stool softeners appropriately. Bowel evacuation techniques: family members learn to assist patients with basic bowel training, including scheduled toileting and the use of aids such as enemas or digital stimulation. Complication recognition and management: training family members to identify and manage common complications such as constipation and bloating, while also providing emotional support for the patient. Collaboration with Healthcare Providers: family members are encouraged to work closely with healthcare professionals, providing feedback on the patient’s care and helping to optimize the bowel management plan (19).

During hospitalization, the experimental group received identical intervention protocols as the control group. Post-discharge management diverged through implementation of our family-involved management protocol. To ensure tiered strategy execution, caregivers were stratified by educational attainment into three competency-based tiers: elementary-level caregivers (primary/lower secondary education or below): focused on mastering essential care tasks to ensure fulfillment of basic daily needs and mitigate care-related complications. Intermediate-level caregivers (upper secondary/vocational education): trained to implement standardized gastrointestinal management protocols targeting prevalent issues like constipation and diarrhea. Advanced-level caregivers (tertiary education or above): equipped to deliver personalized long-term care through comprehensive management of complex clinical presentations.

### 2.2 Content of the management schemes for each level

#### 2.2.1 Elementary-level intervention protocol

Caregivers with lower educational attainment typically demonstrate limited comprehension of medical terminology and caregiving techniques. The management protocol was specifically designed to enhance learning efficacy through intuitive instructional modalities, prioritizing visual aids (illustrated manuals, instructional videos) and hands-on demonstrations. Key components included: ① Daily activity support: provision of graphic-rich educational materials and video tutorials covering essential tasks: positioning, hygiene maintenance, toileting assistance, and dressing support. ② Skin integrity management: structured training in pressure injury prevention, with emphasis on perianal care techniques. ③ Constipation mitigation: implementation of dietary modifications and hydration protocols to regulate bowel motility (20, 21). ④ Bowel habit standardization: simplified guidance for maintaining bowel diaries and establishing defecation routines. ⑤ Health monitoring: user-friendly tracking sheets for documenting dietary intake, bowel patterns, and vital signs to facilitate remote clinical evaluation. The elementary-level intervention protocol is designed to empower caregivers with the capacity to independently master and perform fundamental care tasks—including activities of daily living (ADLs) and skin integrity maintenance—while achieving sustained reductions in constipation incidence through protocolized bowel management, ultimately ensuring patients’ basic care needs are met and care-related complications are minimized.

#### 2.2.2 Intermediate-level intervention protocol

Targeting caregivers with foundational caregiving experience and intermediate educational attainment, this protocol enhanced clinical competencies in managing complex presentations through structured skill development: ① bowel regimen implementation: hands-on training in abdominal massage techniques, anal reflex stimulation, and rectal stimulation protocols to establish defecation routines. Dietary optimization strategies: fiber modulation, fluid intake calibration, and judicious laxative use. ② Symptom-specific nutritional guidance: customized meal planning to prevent paradoxical constipation/bloating through controlled soluble/insoluble fiber ratios. ③ Clinical surveillance system: standardized documentation of bowel patterns and nutritional intake using validated tracking instruments. Protocolized escalation pathways for abnormal symptom recognition and interdisciplinary communication. The intermediate-level intervention protocol was designed to equip caregivers with the capacity to independently perform advanced care techniques such as abdominal massage and bowel training regimens, while adaptively modifying care plans according to individualized patient assessments and proactively detecting clinical anomalies through structured health surveillance systems, thereby establishing competency in managing complex symptomatology and delivering personalized care.

#### 2.2.3 Advanced-level intervention protocol

Designed for caregivers with tertiary education, this protocol emphasized mastery of specialized skills for managing complex presentations and developing individualized long-term care strategies. Key components included: ① expert technique training: competency-based instruction in advanced bowel management methods: anorectal stimulation, manual evacuation protocols, and biofeedback-assisted defecation training. ② Personalized care planning: multidisciplinary care plan formulation integrating daily care protocols, pharmacotherapy oversight, nutrient-dense diet design, and neurogenic bowel-specific physiotherapy. ③ Predictive health monitoring: longitudinal tracking of gastrointestinal motility metrics, body composition trends, and bowel diary analytics with care plan adjustments. The advanced protocol is designed to achieve the following anticipated outcomes: caregivers will attain proficiency in advanced nursing skills, independently manage complex gastrointestinal complications, formulate and adapt personalized care regimens, and ensure continuity in long-term health management through integrated monitoring systems and feedback mechanisms.

This study comprehensively evaluated the management effects of neurogenic bowel dysfunction (NBD) in patients with spinal cord injury using multiple outcome measures. The evaluation focused primarily on improvements in patient symptoms, caregiver execution of care tasks, caregiver burden, and patient quality of life. Patient gastrointestinal function was measured using the Neurogenic Bowel Dysfunction (NBD) score, with comparisons made between the scores at discharge, 6 months post-discharge, and 12 months post-discharge. Caregiver performance was assessed using an execution status form designed by our research team. Allocation concealment was achieved through sequentially numbered, sealed, opaque envelopes. Outcome assessors were blinded to group assignment throughout the 12-month follow-up period. The Caregiver Execution Status Assessment Tool was validated through expert panel review (*n* = 8 rehabilitation specialists with ≥10 years SCI experience; Content Validity Index ≥0.85 for all items). Pilot testing (*n* = 30 caregiver-patient dyads) demonstrated: internal consistency Cronbach’s *α* = 0.89 (95% CI: 0.85–0.92), inter-rater reliability ICC = 0.91 (95% CI: 0.84–0.95), test–retest reliability ICC = 0.87 (95% CI: 0.78–0.93), and construct validity through correlation with NBD outcomes (*r* = −0.64, *p* < 0.001). Five care domains (Bowel Management, Dietary Record, Skin Care, Emotional Support, Health Monitoring) are each scored 0–5: 0 = not performed at all, 1 = very poor execution, 2 = poor execution with major errors/omissions, 3 = satisfactory execution (meets basic requirements), 4 = good execution (task completed accurately and timely), 5 = excellent execution (exceeds expected standards). Total scores range 0–25; average score ≥3 across domains indicates adequate competence for home-based NBD management. While caregiver burden was evaluated using the Zarit Burden Interview (ZBI). Patient quality of life was quantified using the SF-36 Health Survey. For data analysis, paired sample *t*-tests and independent sample *t*-tests were used to compare changes pre- and post-intervention; if the data did not conform to normality, non-parametric tests (Wilcoxon signed-rank test or Mann–Whitney *U* test) were employed. Additionally, to further assess the intervention effects, effect sizes (Cohen’s *d*) were calculated for the numerical comparisons.

## 3 Result

### 3.1 Participant characteristics

Between May 2023 and April 2024, 207 patients were screened and 137 (66.2%) enrolled following stratified randomisation: 69 to routine follow-up and 68 to the tiered intervention. No participants were lost to follow-up. Baseline characteristics were well balanced across all three educational strata (Tables 2–4), with no significant between-group differences in age, sex distribution, ASIA grade, or NBD score (all *p* > 0.05).

**Table 2:** High education level.

Outcome measures	EG (*n* = 8)	CG (*n* = 9)	Cohens’ *d*	*p*-value
Age	42 ± 9	41 ± 8		0.87
Sex (F/M)	4/4	4/5		1.0
ASIA (B/C)	3/5	4/5		1.0
Baseline NBD	19.5 ± 5.8	19.3 ± 6.1		0.95
6-months NBD	15.5 ± 4.5	16.4 ± 5.1	−0.19 (−0.71,0.33)	0.71
12-months NBD	14.0 ± 3.9	14.2 ± 4.3	−0.05 (−0.57,0.48)	0.92
6-months exec. status	4.2 ± 0.2	4.0 ± 0.4	0.63 (0.10, 1.16)	0.21
12-months exec. status	4.5 ± 0.3	4.2 ± 0.5	0.71 (0.18, 1.2)	0.15
6-months ZBI score	47 ± 6	50 ± 8	−0.42 (−0.94,0.10)	0.39
12-months ZBI score	45 ± 8	48 ± 7	−0.40 (−0.92,0.12)	0.43
Baseline SF-36 score	47 ± 3	46 ± 6	0.21 (−0.31,0.73)	0.67
12-months SF-36 score	61 ± 6	59 ± 8	0.28 (−0.24, 0.80)	0.57

*p < 0.05. EG, Experimental group; CG, Control group; Cohen’s d, Effect size; *p*-value, Statistical significance.

**Table 3:** Med education level.

Outcome measures	EG (*n* = 20)	CG (*n* = 20)	Cohens’ *d*	*p*-value
Age	41 ± 7	40 ± 4		0.58
Sex (F/M)	8/12	10/10		0.75
ASIA (B/C)	7/13	6/14		1.0
Baseline NBD	21.8 ± 5.2	22.0 ± 5.7	−0.04 (−0.68, 0.60)	0.91
6-months NBD	17.6 ± 4.8	19.2 ± 5.4	−0.48 (−0.96, 0.33)	0.33
12-months NBD	16.8 ± 4.6	18.25 ± 5.4	−0.29 (−0.93, 0.36)	0.37
6-months exec. status	3.6 ± 0.3	3.4 ± 0.5	0.49 (−0.17, 1.14)	0.13
12-months exec. status	4.2 ± 0.4	3.8 ± 0.6	0.78 (0.12, 1.45)	0.02*
6-months ZBI score	46 ± 7	50 ± 6	−0.61 (−1.27, 0.04)	0.06
12-months ZBI score	42 ± 5	45 ± 8	−0.45 (−1.10, 0.20)	0.17
Baseline SF-36 score	48 ± 4	47 ± 6	0.20 (−0.45, 0.84)	0.54
12-months SF-36 score	53 ± 7	50 ± 6	0.46 (−0.20, 1.11)	0.15

*p < 0.05. EG, Experimental group; CG, Control group; Cohen’s d, Effect size; *p*-value, Statistical significance.

**Table 4:** Low education level.

Outcome measures	EG (*n* = 40)	CG (*n* = 40)	Cohens’ *d*	*p*-value
Age	39 ± 3	38 ± 3		0.75
Sex (F/M)	21/19	20/20		1.0
ASIA (B/C)	10/30	9/31		1.0
Baseline NBD	20.5 ± 5.5	21.8 ± 6.0	−0.23 (−0.68, 0.22)	0.32
6-months NBD	18.0 ± 5.0	20.5 ± 5.4	−0.48 (−0.93, −0.03)	0.04*
12-months NBD	17.0 ± 4.8	19.2 ± 5.2	−0.44 (−0.89, 0.01)	0.05*
6-months exec. status	3.4 ± 0.3	3.0 ± 0.3	1.33 (0.84, 1.82)	<0.01*
12-months exec. status	4.0 ± 0.4	3.3 ± 0.5	1.55 (1.04, 2.06)	<0.01*
6-months ZBI score	42 ± 8	52 ± 7	−1.33 (−1.82, −0.84)	<0.01*
12-months ZBI score	39 ± 7	50 ± 8	−1.46 (−1.96, −0.96)	<0.01*
Baseline SF-36 score	45 ± 6	44 ± 6	0.17 (−0.28, 0.62)	0.46
12-months SF-36 score	52 ± 4	48 ± 5	0.88 (0.41, 1.35)	<0.01*

*p < 0.05. EG, Experimental group; CG, Control group; Cohen’s d, Effect size; *p*-value, Statistical significance.

### 3.2 Neurogenic bowel dysfunction outcomes

Intervention effects on NBD scores varied substantially by caregiver educational attainment. Among caregivers with tertiary education (*n* = 17), both groups showed comparable improvements over 12 months with no significant between-group differences at any timepoint (*p* = 0.92; Cohen’s *d* = −0.05; Table 2). The secondary-education cohort (*n* = 40) demonstrated numerically favorable but non-significant trends toward the intervention group at 12 months (*p* = 0.37; *d* = −0.29; Table 3). By contrast, caregivers with primary education only (*n* = 80) achieved significantly greater NBD reductions in the intervention arm at both 6 and 12 months (*p* = 0.04 and *p* = 0.05, respectively; *d* = −0.44 to −0.48; Table 4), with benefits sustained throughout follow-up.

### 3.3 Caregiver competency and burden

Execution status scores improved significantly only in lower-education strata. The primary-education group demonstrated large effect sizes at both assessments (*d* = 1.33 and 1.55; both *p* < 0.01), while the secondary-education cohort showed a modest but significant improvement at 12 months (*d* = 0.78; *p* = 0.02). No significant differences emerged in the tertiary-education group.

Caregiver burden, measured by the Zarit Burden Interview, decreased significantly in the primary-education subgroup at both 6 and 12 months (both *p* < 0.01; *d* = −1.33 to −1.46), representing clinically meaningful reductions. No burden relief was detected in higher-education strata.

### 3.4 Quality of life

SF-36 total scores at 12 months improved significantly only in the primary-education cohort (*p* < 0.01; *d* = 0.88), with the magnitude exceeding the minimal clinically important difference. No quality-of-life gains were observed in secondary or tertiary education groups.

### 3.5 Educational gradient in treatment response

A clear dose–response relationship emerged between caregiver educational attainment and intervention benefit. Across four primary endpoints assessed at two timepoints (eight outcome measures), the proportion achieving statistical significance increased stepwise from zero of eight in the tertiary-education stratum to one of eight in the secondary-education stratum to seven of eight in the primary-education stratum. Peak effect sizes similarly escalated inversely with educational level, with magnitudes in the lowest-education cohort consistently exceeding thresholds for large clinical significance.

## 4 Discussion

This randomised, stratified trial is, to our knowledge, the first to link caregiver educational attainment to the magnitude of benefit derived from a tiered family-involved management programme for neurogenic bowel dysfunction (NBD) after thoracic spinal cord injury. The findings show that caregivers with lower formal education experienced the greatest improvement in patient bowel function, caregiving proficiency, caregiver burden and patient quality of life, whereas those with tertiary education derived little additional benefit beyond routine follow-up.

Several mechanisms may underlie this gradient. First, caregivers with limited educational backgrounds typically have restricted baseline health literacy; the visual, skills-oriented materials and hands-on coaching contained in the elementary-level module therefore closed a larger knowledge gap than in higher-educated peers. Second, higher-educated caregivers may already employ adaptive coping strategies or access external resources, leaving less room for incremental gain. Third, behavioral-change theory suggests that simple, concrete guidance is more likely to be adopted when self-efficacy is initially low, consistent with the large effect sizes observed in the primary-education cohort (14, 15).

While family-centered rehabilitation has been established for stroke and neurological disorders (22, 23), literacy-stratified interventions for NBD management represent a novel implementation. Previous caregiver education programs have employed uniform content delivery (11, 19), whereas our tiered framework differentially allocates instructional complexity—visual aids and task-focused training for lower-educated caregivers versus advanced problem-solving for higher-educated groups. This adaptive design builds upon health literacy frameworks (24) and tiered self-management models in chronic disease (25) but, to our knowledge, has not been applied to neurogenic bowel dysfunction. The pronounced benefit gradient observed validates this approach’s theoretical foundation and practical utility.”

Clinically, these data argue for preferential allocation of training resources to families with lower educational attainment, where return on investment is greatest. Programmes aimed at secondary-educated caregivers should emphasise early-phase reinforcement to prevent attenuation of benefit, while for tertiary-educated caregivers, future interventions might focus on advanced problem-solving or digital decision-support rather than basic skills training.

This study has several limitations. First, the tertiary-education subgroup was small (*n* = 17), limiting statistical power and precision of effect estimates. Second, several unmeasured confounders warrant consideration. Prior caregiving experience or healthcare-related occupational backgrounds could independently enhance care execution, while socioeconomic status may affect access to supplementary resources (e.g., private consultations, assistive equipment). Additionally, baseline health literacy—distinct from formal education—and social support networks (family structure, community resources) likely modulate intervention uptake and sustainability. These variables, not systematically assessed in our study, may partially explain the observed educational gradient and should be incorporated into future predictive models. Third, the decision not to register this trial prospectively in a clinical registry—while consistent with exemption criteria for educational interventions—may limit discoverability and transparency. Future studies should consider registration to align with evolving best practices in trial reporting. Fourth, follow-up was limited to 12 months; longer observation is required to assess sustainability of benefits and late complications. Finally, the single-center design may limit generalizability to other healthcare systems or cultural contexts.

Future research should employ multicentre designs with larger, balanced strata and consider composite indices of caregiver capacity. Exploring e-learning platforms, adaptive feedback loops and peer-support networks may further enhance intervention scalability.

In summary, a literacy-adaptive, family-centred management strategy yields clinically meaningful, durable improvements in NBD outcomes, particularly among caregivers with primary or secondary education. Tailoring post-discharge rehabilitation to caregiver educational level represents a pragmatic pathway to maximise functional recovery and quality of life after spinal cord injury.

## 5 Conclusion

This prospective stratified randomised trial shows that a literacy-adaptive, family-centred programme for neurogenic bowel dysfunction after thoracic spinal cord injury yields meaningful and sustained benefits, particularly for caregivers with primary or secondary education. Over 12 months, the tailored intervention produced larger reductions in NBD scores and caregiver burden and greater gains in caregiving execution and SF-36 quality of life than guideline-based routine care, whereas caregivers with tertiary education derived only marginal additional benefit, suggesting a ceiling effect.

These findings suggest that educational resources should be prioritised for families with lower formal education, using visual, skills-oriented materials and hands-on coaching, while secondary-educated caregivers may need structured reinforcement and tertiary-educated caregivers more advanced decision-support rather than basic skills training. Stratifying post-discharge care by caregiver competence is therefore a practical way to maximise functional recovery and reduce long-term health-system burden. Interpretation is limited by the small tertiary-education subgroup, unmeasured factors such as prior healthcare experience and socioeconomic status, and a 12-month, single-centre follow-up.

Overall, matching the depth and delivery of neurogenic bowel management education to caregiver literacy is both feasible and effective. For health systems—particularly in low- and middle-income settings—literacy-adaptive discharge protocols could optimise use of low-cost materials, prevent NBD-related complications and readmissions, reduce inequities in home care, and provide a template for managing other chronic complications of spinal cord injury at scale.

## Data Availability

The raw data cannot be made publicly available due to hospital ethics policies and patient confidentiality requirements. Data inquiries may be directed to the corresponding author.
